# Identifying Issue Frames in Text

**DOI:** 10.1371/journal.pone.0069185

**Published:** 2013-07-16

**Authors:** Eyal Sagi, Daniel Diermeier, Stefan Kaufmann

**Affiliations:** 1 Kellogg School of Management, Northwestern University, Evanston, Illinois, United States of America; 2 Department of Linguistics, Northwestern University, Evanston, Illinois, United States of America; University of Maryland, College Park, United States of America

## Abstract

Framing, the effect of context on cognitive processes, is a prominent topic of research in psychology and public opinion research. Research on framing has traditionally relied on controlled experiments and manually annotated document collections. In this paper we present a method that allows for quantifying the relative strengths of competing linguistic frames based on corpus analysis. This method requires little human intervention and can therefore be efficiently applied to large bodies of text. We demonstrate its effectiveness by tracking changes in the framing of *terror* over time and comparing the framing of *abortion* by Democrats and Republicans in the U.S.

## Introduction

Psychologists and social scientists have long observed that the way in which a question or problem is presented to people can impact their attitudes and decisions [1]. *Framing* is a widely discussed instance of this phenomenon: The choice of words and metaphors in talking about a given issue can affect recipients’ interpretations and biases, making some actions or strategies appear more plausible than others [2]. Psychologically, framing relies on existing associative relationships between the words and on the ability of the audience to tacitly “flesh out” what is literally said. The exploration of the mental representations involved in these processes is an active and long-standing topic of research in cognitive psychology [2].

Frames are particularly important in shaping public opinion [3,4,5]. For example, an estate tax can be framed as double-taxation or as redistributive tax [5], the death penalty can be discussed in the context of morality frames (“an eye for an eye” versus “thou shalt not kill”), constitutionality frames (“cruel and unusual punishment” versus “justice is served”), or fairness frames (“wrongful execution” vs. “acceptable price to pay”) [6]. Political science research has established that successful issue frames influence public opinion [5], and that policy makers respond to shifts in public opinion [7,8].

One approach to the study of issue framing has been the use of controlled experiments with human subjects [5]. Another approach has tried to identify issue frames in text corpora, such as congressional records or newspaper coverage. Researchers interested in identifying issue frames from text frequently rely on manually annotated document collections [9,10]. The production of such annotations is slow, labor-intensive, and dependent on the judgments of experts. It does not lend itself easily to the rapid analyses of large data sets. However, as the amount of textual data available in electronic form has been rapidly increasing in recent years, the demand for tools to facilitate fast and efficient analyses of large data sets has risen dramatically. To meet this demand, researchers have turned to machine-learning methods from computational linguistics that were originally developed for applications in other areas of text analysis, such topic identification [11,12] and opinion classification [13,14].

Most of these methods rely to some extent on word co-occurrence patterns. It is commonly assumed in language technology and computational linguistics that a word’s co-occurrence patterns with other words provide a readily observable approximation of its semantic content [15,16]. A prominent example is Latent Semantic Analysis (LSA) [17,18], which has been applied to a wide range of tasks, including word sense discrimination [19,20], text summarization [21], automatic scoring of student essays [22], and identifying semantic change [23]. In this paper we present an LSA-based approach designed to observe and quantify variation in the framing of concepts across time or speaker/author populations. This method is designed to facilitate the analysis of frames in large corpora. However, while it allows for rapid large-scale statistical analysis, our approach does not replace other, complementary, methodologies that offer a more in-depth exploration of the data. In effect, this paper describes a new statistical tool that researchers can use to test the validity of their hypotheses. We illustrate this method by applying it to two examples of framing in political debates in the US senate: the rise and time-course of the framing of *terror* as a military struggle following the events of September 11^th^, 2001, and the different framings of *abortion* by Democrats and Republicans. In the latter case, we also compare the framing of abortion in the US Senate to that found in a major US newspaper – The New York Times.

Analyzing debates in the US Senate allows us to assess how issue frames are correlated with party affiliation, as well as the prevalence of certain frames over time. Previous work has demonstrated that the content of speeches by U.S. Senators is highly correlated with party affiliation [13] and ideological positioning [14]. Previous research has also demonstrated the impact of media frames on public opinion formation [6,24], which we can explore by analyzing framing in the New York Times.

Underlying our application of LSA is the hypothesis that the possible framings of a concept manifest themselves in ways that are analogous to those of the different senses of an ambiguous word. The task of frame identification is therefore akin to that of word sense disambiguation. Just as the intended sense of a given word occurrence can usually be determined by inspection of the text surrounding it, the framing of a concept will be discernible through the terms with which it is used. For instance, if the word *terror* is framed as a criminal act, then the terms in the vicinity of its occurrences will tend to be associated with words like *justice*, *arrest*, and *trial*. In contrast, if *terror* is framed as a military struggle, it is more likely to co-occur with terms that are associated with *fight, win*, and *war*. By observing such patterns across a large body of text, we can track changes in framing across time and assess the impact of other variables, such as the speaker’s party affiliation.

### Analyzing Framing

Frames, like word meanings, are complex psychological entities that are difficult to identify. However, if the contexts in which a word occurs provide some information about its framing, we can exploit that information by exploring the distances or similarities of those contexts vis-à-vis those of certain manually selected words which we take to be prototypically associated with particular frames. For instance, if terror is framed as an act of war rather than a crime, then, on average, the contexts in which the word *terror* occurs should be more similar to the contexts in which the word *war* occurs than to those in which the word *crime* occurs. This is the assumption at the core of the method we present in this paper. More specifically, we assume that the semantic content of a word can be approximated by observing the words that it frequently co-occurs with [15,16].

**Figure 1:**
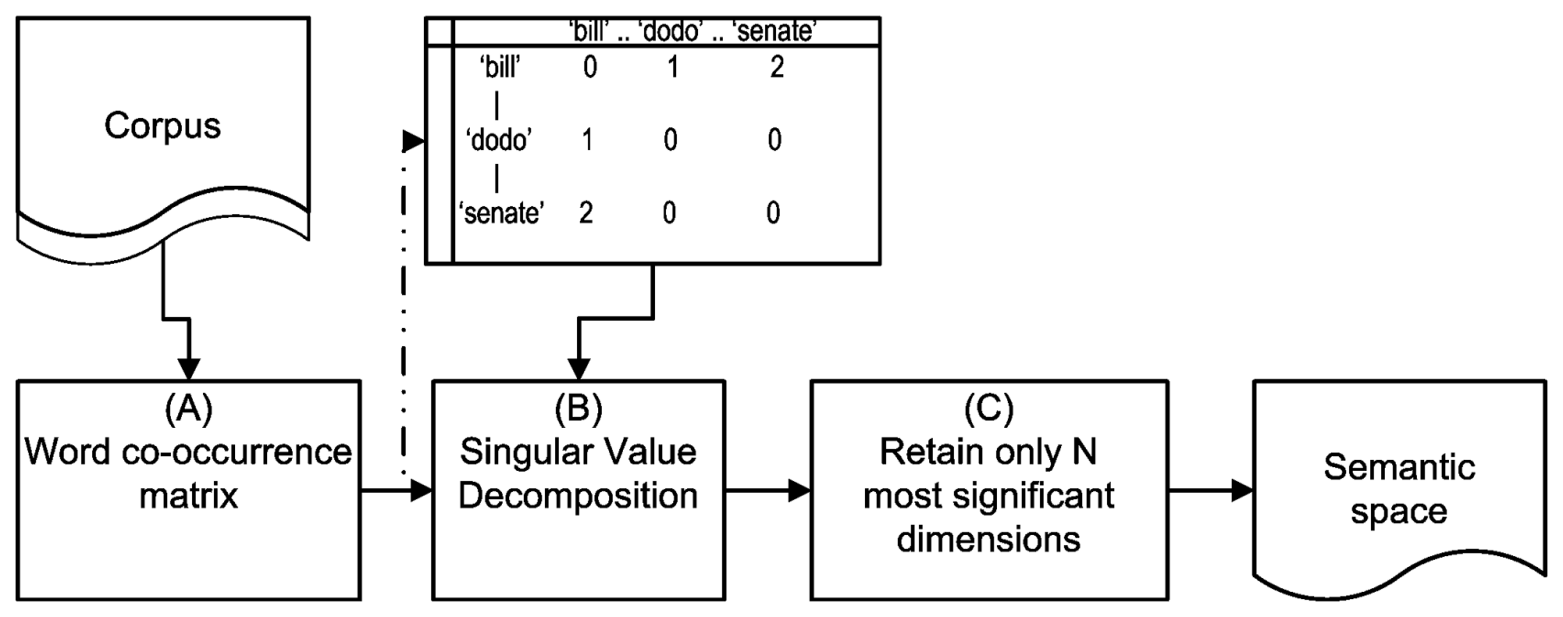
A schematic diagram describing the computation of a semantic space. This figure describes the process of generating a semantic space following the *Wordspace* paradigm. (A) In the first step an NxM matrix of word co-occurrence is computed. The words for this matrix are chosen based on their frequency of occurrence. (B) Singular Value Decomposition (SVD; a generalized form of factor analysis) is performed on the matrix. This transformation results in a high-dimensional space. (C) Finally, the least significant dimensions of the matrix are dropped so that only the most important, content bearing, dimensions are part of the semantic space. By default, the implementation of *Wordspace* we used, Infomap, retains 100 dimensions.

**Figure 2:**
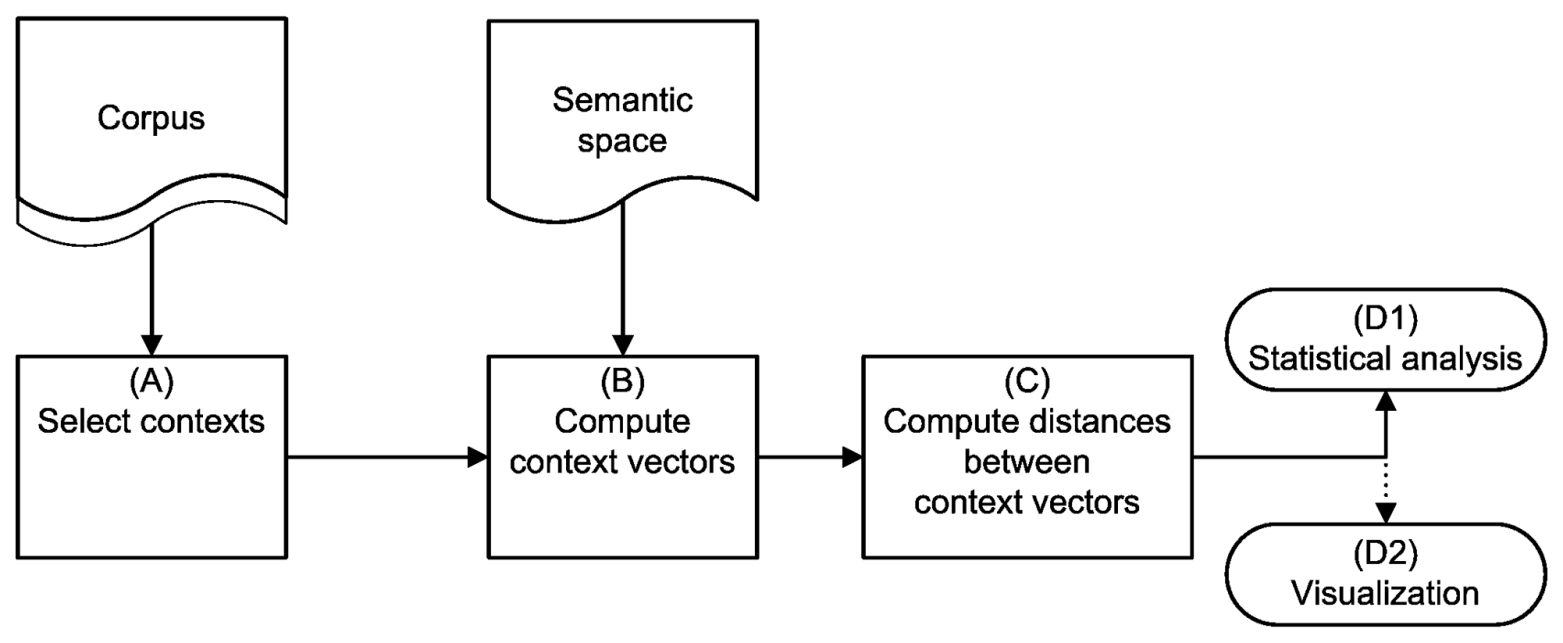
A schematic diagram of the method presented in this paper. The method we present in this paper is comprised of 4 distinct steps. (A) In the first step an appropriate set of contexts is selected from a corpus based on keywords that represent the target word and its possible frames. (B) Next, a vector is computed for each context using vector addition. The vectors for each word are provided by a pre-computed semantic space. (C) In the third step, distances are computed between related groups of context vectors (i.e., the target word and its possible frames). (D) Finally, a statistical analysis (D1) and an optional visualization (D2) are performed on the resulting distances.

LSA is a collective term for a family of methods aimed at operationalizing this intuition by deriving a measure of similarity between words from their co-occurrence behavior in a collection of documents. Technically, words are associated with vectors in a high-dimensional space. The most commonly used measure in this framework is the cosine between the associated vectors: A high cosine (i.e., low angle) indicates positively correlated co-occurrence profiles, hence by assumption semantic similarity. Within the LSA family, our method is based on the *Wordspace* paradigm [18,25] (see Figure 1 for a general overview of how a semantic space is generated within the paradigm. Text S1 provides details and differences with other LSA-style methods). Figure 2 presents a schematic outline of our method of analysis, as described in more detail below.

Since we are interested in the individual occurrences (tokens) of a given word (type), we use the word vectors to derive *context vectors* for all occurrences of the target word (such as *terror* or *abortion*). Formally, the context vector for a given token is the normalized sum of the word vectors associated with the words surrounding it within a text window of a certain fixed width. A similar method was first applied in word-sense discrimination [20]. Analogously, we hypothesize that differences in framing of a given word can be observed and measured in terms of the context vectors of its occurrences. To this end, the collection of context vectors for all occurrences of the target word can be divided up along various independent variables. Furthermore, because vector similarities are represented as simple scalars, differences in framing can be explored using standard methods for statistical hypothesis testing (e.g., by computing a *t*-statistic comparing the distances between context vectors representing uses of *terror* and *war* before and after September 11^th^, 2001).

Although the context vectors we compare are associated with concrete words like *terror* and *war*, our similarity measure is different from a simple co-occurrence count or other measures of collocational strength. What we are interested in is a relationship between regions in a densely populated vector space. The particular words we choose to represent frames -*war* in our example – are merely convenient identifiers for such regions. Consequently, our analysis of the framing of *terror* yields qualitatively similar results whether the framing term is *war*, *fight*, or *military*, even though the pattern of direct co-occurrence of the term *terror* with these words varies considerably.

## Materials and Methods

### The Corpora

The first corpus we use is intended to track changes in issue frames among the debates of policy elites, here U.S. Senators. It includes transcripts of all speeches given on the floor of the U.S. Senate from 1989 to June of 2006 [13,14]. It is composed of 229,527 speeches totaling over 125 million transcribed words.

Our second corpus can be used to identify issue frames in the mass media and observe their development over time. That corpus is a collection of New York Times articles from 1987 to 2007, available from the Linguistic Data Consortium [26]. It is comprised of 1,855,658 articles totaling over 4 billion words.

### Methods

We prepared the corpus for analysis using Infomap [27] to generate a semantic space from the corpus, and derived word vectors for the 20,000 most frequent non-stopwords in the corpus. (Text S1 provides more information on this process.) 

Next, we identified all of the contexts in which the words of interest and their possible frames occur. This resulted in 3,147 contexts for *terror*, 27,863 for *crime*, and 59,686 for *war*, 10,954 for *abortion*, 17,203 for *choice*, 48,665 for *life*, 11,470 for *woman*, and 9,168 for *mother*. In the New York Times analysis, this resulted in 62,561 contexts for *abortion*, 109,682 for *choice*, 566,764 for *life*, 222,623 for *woman*, and 261,562 for *mother*.

For each of these contexts we computed a context vector to be used in the analysis. Context vectors were calculated based on word vectors in a semantic space generated from the same corpus. Each context vector was computed using vector addition over a window of 15 words before and 15 words after the target word, a window size that was chosen because it allows a substantial portion of context to be used. After the application of vector addition the vectors were normalized to a length of 1 by dividing each vector by its length so that the length of the resulting vector would not affect further calculations.

To calculate the semantic distance we averaged the cosines between all of the vectors representing the target term on the one hand, and those representing its possible frames for each year. Importantly, since the first dimension of the vectors resulting from Singular Value Decomposition is always positive and correlated with the frequency of the term, we omitted the first dimension when computing the cosines [28]. Statistical analysis was conducted using ANOVAs.

## Results

### Rise of the War on Terror

Our first example is the framing of *terror* as a *war* after September 11^th^, 2001. While this framing has become highly salient in recent political discourse, Lakoff [29] suggests that initially there was another, competing frame – *terror* as a *crime*. In our analysis we investigated whether *terror* was framed in terms of *war* rather than *crime* after the events of 9/11. Following Lakoff’s analysis, we hypothesized that we should see a significant decrease in the distance between the context vectors of *terror* and *war* after 2001 compared to previous years. In contrast, we have no such expectation in the case of *terror* and *crime*.

**Figure 3:**
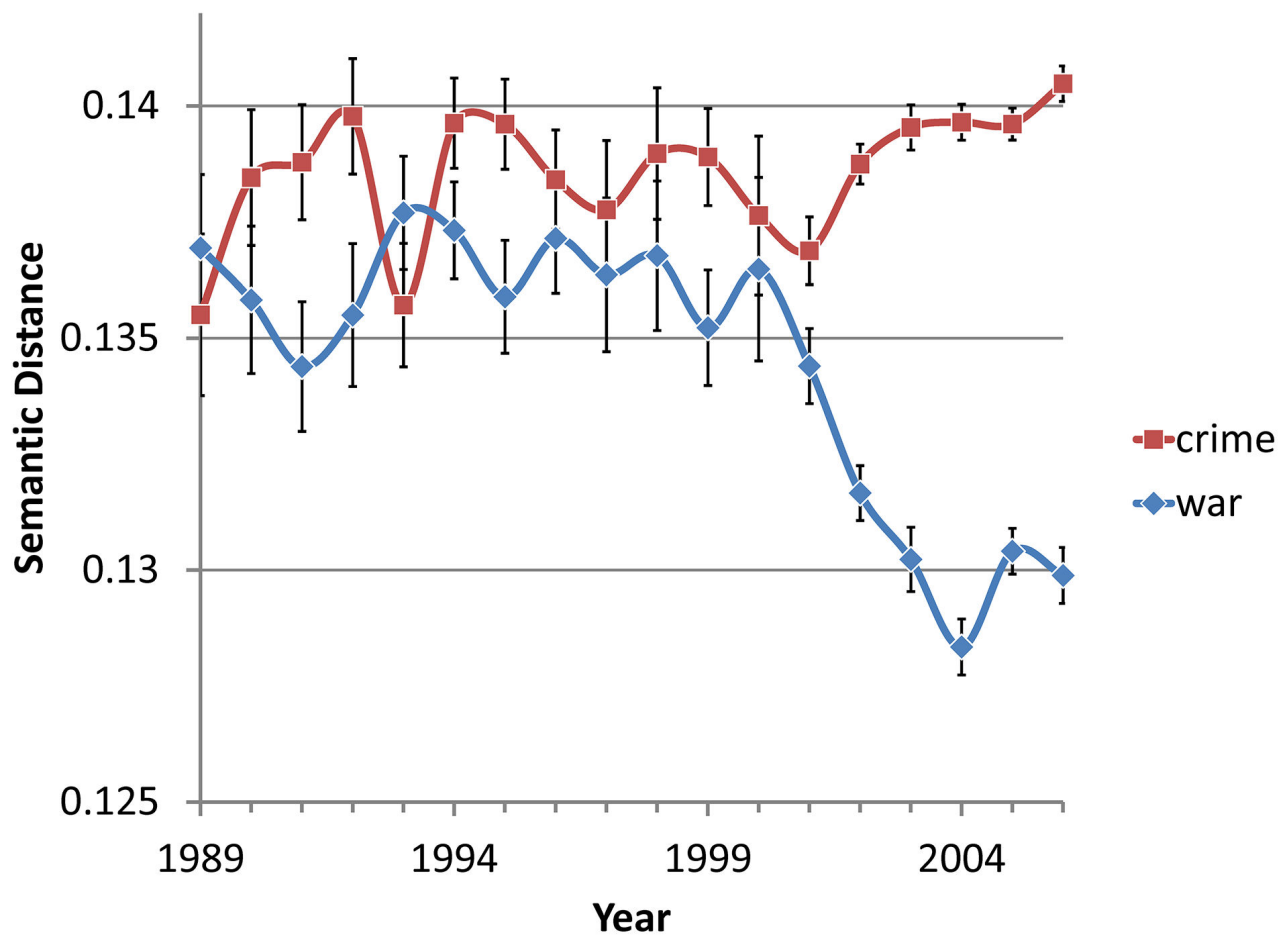
Mean context vector distances of *war* and *crime* from *terror* by year. As the zero point of the graph represents identical uses for the term in question and terror, lower positions in the graph represent greater relevance for the term as a frame of *terror*. Error bars represent standard error.

We computed context vectors for the occurrences of the target word *terror* and its two hypothesized frames *war* and *crime* in our corpus. We then computed the mean distance between *terror* and each of *war* and *crime* for each year (Figure 3). As predicted, the distance between the vectors for *terror* and *war* is significantly reduced after 2001 (*M*_<2001_ = 0.1363, SD_<2001_ = 0.001, *M*_>2001_ = 0.1301, SD_>2001_ = 0.0001; *F*(1,15) = 127.48, MSE = 0.000001, *p* < .0001, *η* ^2^_*p*_ = .90) while the distance between the vectors for *terror* and *crime* does not show a similar reduction and in fact trends in the opposite direction (*M*_<2001_ = 0.1383, SD_>2001_ = 0.001, *M*_>2001_ = 0.1396, SD_>2001_ = 0.0006; *F*(1,15) = 4.06, MSE = 0.000002, *p* = .062, *η* ^2^_*p*_ = .21). This analysis provides support for the claim that post-9/11 *terror* was increasingly framed in terms of *war* rather than *crime*. Interestingly, it appears that of the two, *war* was the stronger and more prevalent framing of *terror* even prior to 9/11.

**Figure 4:**
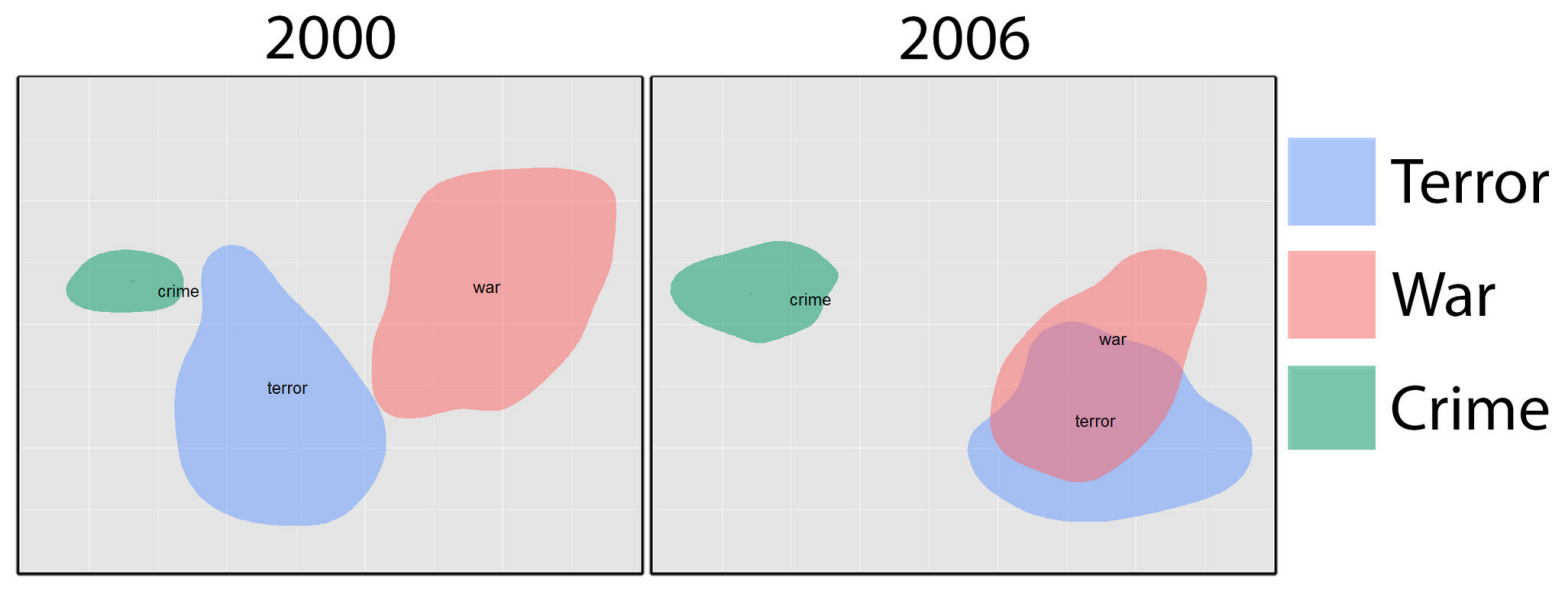
2-dimensional visualization of the context vectors of *war*, *crime*, and *terror*. The distance between points in the visualized space represents the semantic distance between the points. That is, the further apart two regions of space are the more dissimilar they are. Overlap represents an overlap in the contexts of use for the two terms. These figures were extracted from Movie S1. The left frame is for June of 2000, and the right is for June of 2004. The data used in each frame is based on the 12 months starting with the indicated month. Because of memory constraints, context vectors for the 3 terms were aggregated by speaker for each month. This aggregated set of vectors was then used as a whole to compute the MDS, and plotted as a yearly running average on a month-by-month basis.

It is also possible to visualize this space more generally using methods for dimensionality reduction, such as multidimensional scaling (Figure 4; see Movie S1 for a complete set in movie format). We chose to use multidimensional scaling for the visualization because it focuses on maintaining the relative distances between points in the space. This makes it particularly suitable for spatial visualization. Nevertheless, there are many other options for dimensionality reduction, including factoring methods such as the one we used to generate the semantic space (SVD).

### The Abortion Debate

**Figure 5:**
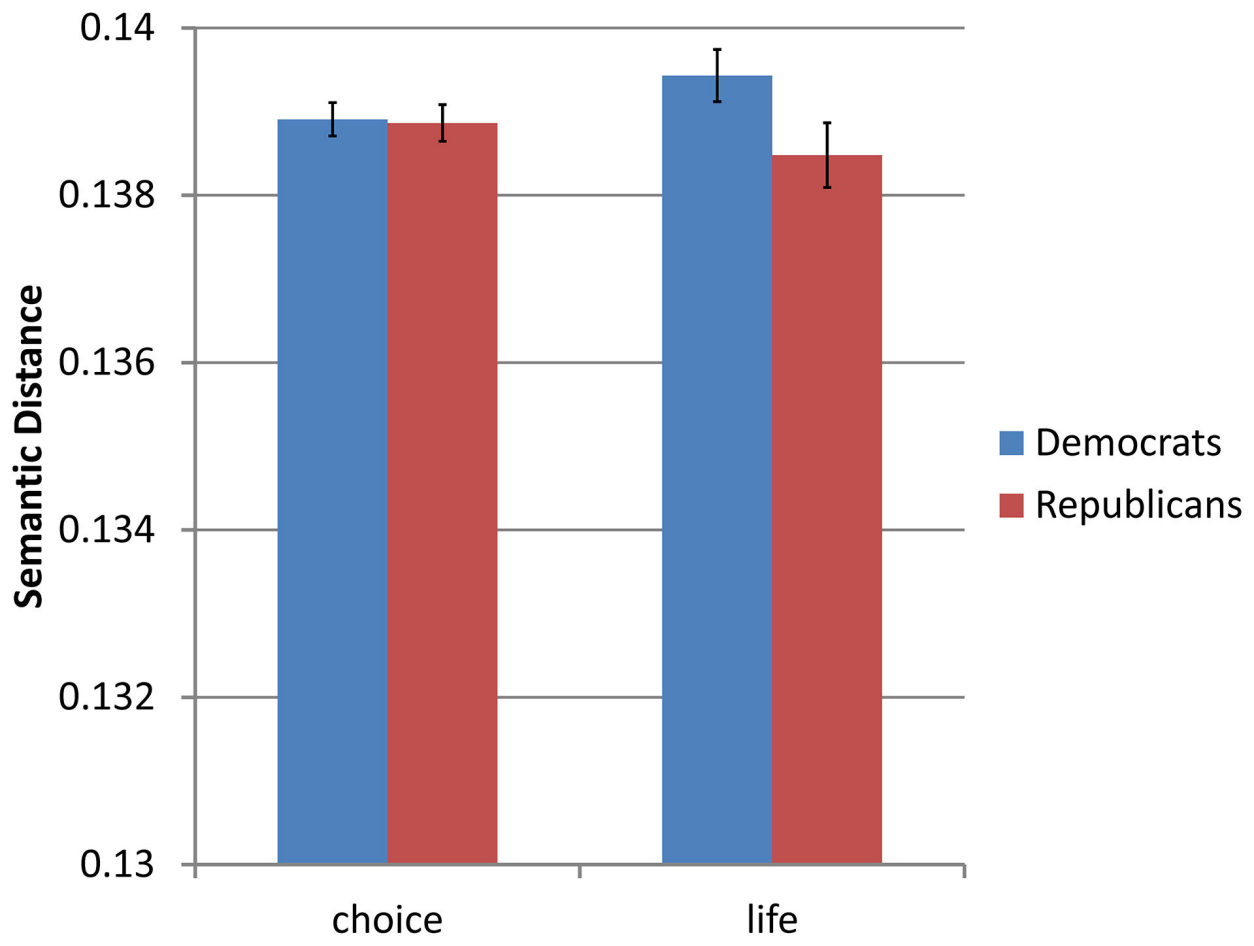
Mean context vector distances of *choice* and *life* from *abortion* by the speaker’s party affiliation. As the zero point of the graph represents identical uses for the term in question and abortion, lower positions in the graph represent greater relevance for the term as a frame of *abortion*. Error bars represent standard error.

The second example concerns the political debate on abortion in the US. Whereas the framing of *terror* as war became close to universal in the US political debate, especially after 2001, the topic of abortion is one which continues to polarize the political arena. The two major positions in this debate are often labeled *pro-choice* (mostly Democrats) and *pro-life* (mostly Republicans). While there are many possible terms that might be used to frame this debate, we chose to focus on the terms *choice* and *life*. The first represents the notion that an abortion should be primarily viewed through the lens of a woman’s right to choose (a position usually preferred by Democrats) while the second frames the debate in terms of the consequences to the fetus’ “right to life” (and should therefore be a frame preferred by Republicans). The mean distances by party (Figure 5) reflect this difference in position between the parties (‘choice’: *M*_*D*_ = 0.1389, *SD*_*D*_ = 0.001, *M*_*R*_ = 0.1388, *SD*_*R*_ = 0.001; ‘life’: *M*_*D*_ = 0.1394, *SD*_*D*_ = 0.001, *M*_*R*_ = 0.1385, *SD*_*R*_ = 0.002). As predicted, there is a significant interaction between the party and frame used (*F*(1,17) = 17.91, MSE = 0.0000002, *p* = < .001, *η* ^2^_*p*_ = .51).

**Figure 6:**
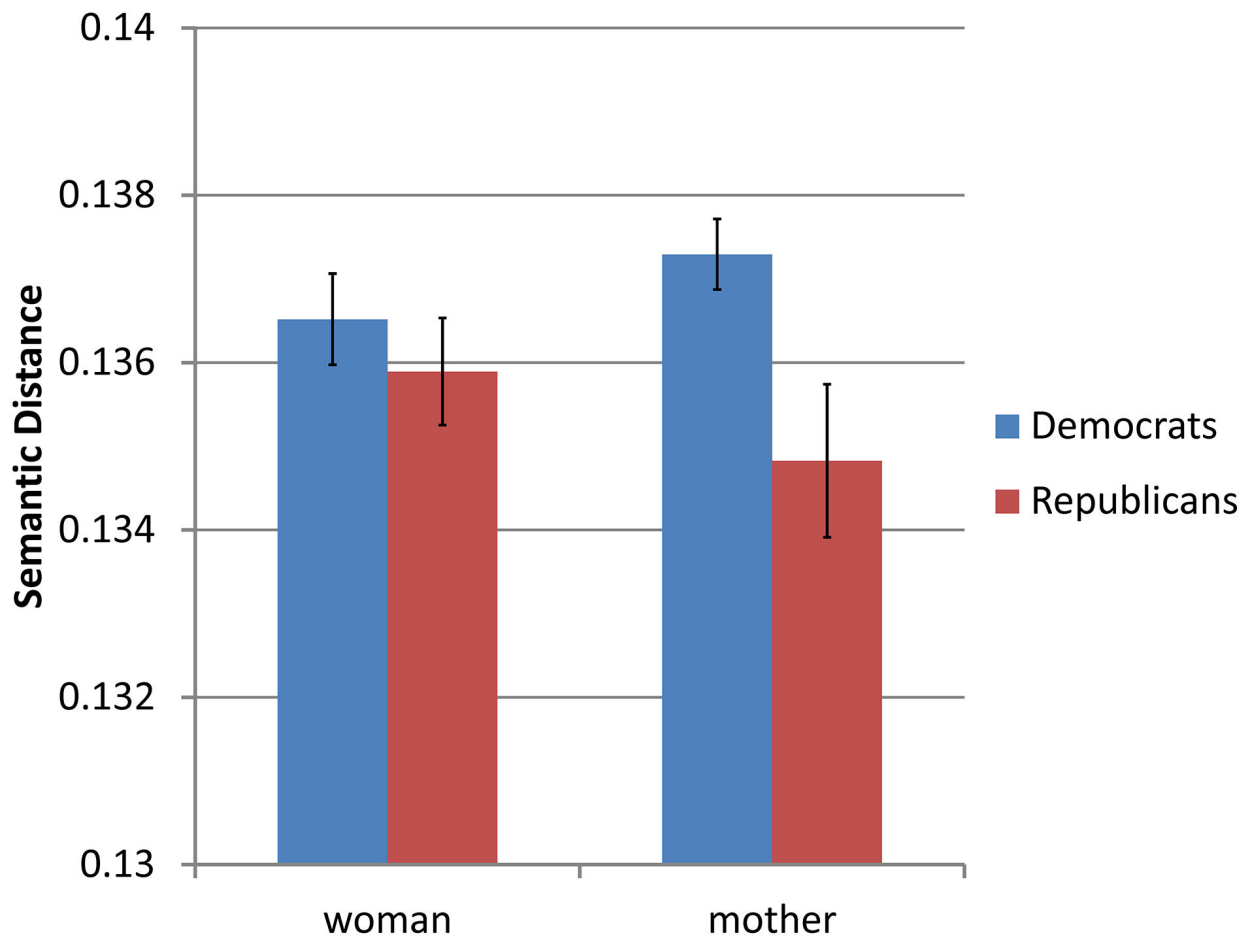
Mean context vector distances of *mother* and *woman* from *abortion* by the speaker’s party affiliation. As the zero point of the graph represents identical uses for the term in question and abortion, lower positions in the graph represent greater relevance for the term as a frame of *abortion*. Error bars represent standard error.

The difference in framing should also be evident in the ways in which the parties refer to women undergoing the procedure [30]. That is, a focus on the effects of the procedure on the baby might be strengthened by referring to such individuals as *mothers*. In contrast, the term *woman* is more neutral with regards to the procedure. We therefore predicted that the framing of abortion by Republicans will be closer to their use of the term *mother* than for Democrats, but that no such differences will be found with regards to the term *woman*. The mean distances by party (Figure 6) support this prediction – Democrats are less likely to speak about abortion using terms associated with *mother* than Republicans (‘mother’: *M*_*D*_ = 0.1373, *SD*_*D*_ = 0.002, *M*_*R*_ = 0.1348, *SD*_*R*_ = 0.004; ‘woman’: *M*_*D*_ = 0.1365, *SD*_*D*_ = 0.002, *M*_*R*_ = 0.1359, *SD*_*R*_ = 0.003; Interaction term: *F*(1,17) = 11.33, MSE = 0.0000013, *p* < .01, *η* ^2^_*p*_ = .40).

In political science, issue frames are generally defined as a focus on a single dimension of an issue [5]. This definition suggests that the two linguistic frames (*choice*/*life* and *woman*/*mother*) are, in actuality, manifestations of a single issue frame. The “pro-choice” frame corresponds to *choice-woman*, the “pro-life” frame to *life-mother*. That is, a preference for framing *abortion* in terms of *life* should also result in a preference to frame it in terms of *mother*. If this is the case, then there should be a positive correlation between the changes that these preferences undergo over time.

We tested this hypothesis by calculating a “frame preference” index for each of the two frames for each party and year. As predicted, the two indices show a significant positive correlation (*r*(35) = .52, *p* < .05). Therefore we can conclude that the two frames are not independent, but rather represent two facets of a single overarching frame of *abortion*.

**Figure 7:**
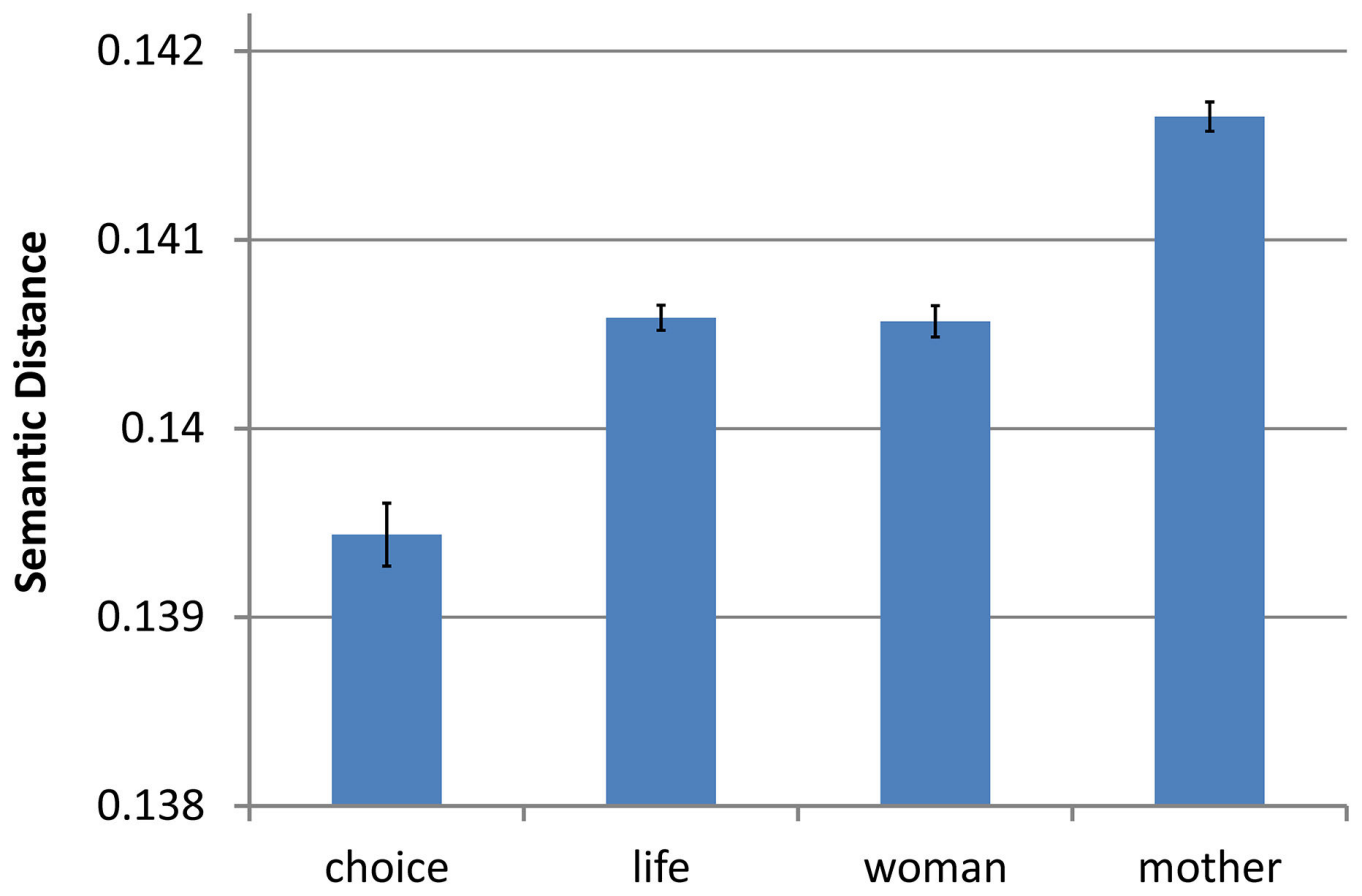
Mean context vector distances of *choice*, *life*, *mother*, and *woman* from *abortion* in the NYT. As the zero point of the graph represents identical uses for the term in question and abortion, lower positions in the graph represent greater relevance for the term as a frame of *abortion*. Data plotted is for all articles appearing in the New York Times for the years 1989-2006. Error bars represent standard error.

There has been an extensive debate over media influence and bias [30]. Experimental research has shown that the media can shape opinion not only through explicit commentary, but also through issue frames [5,24]. We can investigate suggested media framing directly using our method. Here we focus on coverage in the New York Times, which is typically viewed as a relatively liberal newspaper, i.e. leaning towards a “pro-choice” position. Figure 7 presents the mean distances from *abortion* for the 4 terms we explored above. The results provide support for the claim that, at least in the New York Times, journalists frame abortion in terms of choice and are less likely to use terms that imply motherhood in these contexts (‘choice’: *M* = 0.1394, *SD* = 0.0007; ‘life’: *M* = 0.1406, *SD* = 0.0003; *F*(1,17) = 76.84, MSE = 0.0000002, *p*<.0001, *η* ^2^_*p*_ = .82; ‘woman’: *M* = 0.1405, *SD* = 0.0003; ‘mother’: *M* = 0.1417, *SD* = .0003; *F*(1,17) = 241.295, MSE = 0.00000005, *p*<.0001, *η* ^2^_*p*_ = .93). An analysis of the correlation of the frame preference indices similar to that conducted earlier results in a similarly positive correlation (*r*(17) = .50, *p* < .05). This provides further evidence supporting the hypothesis that a single issue frame is underlying the observed preferences for both linguistic frames.

## Discussion

In this paper we described a new method for visualizing and quantifying differences in the framing of terms and conceptual change in textual data. The data acquired by this method is amenable to many types of statistical analyses, from simple hypothesis testing to complex regression models and time series analysis. We hope that this method will be an effective aid for researchers in the use of available texts and enable new types of questions to be answered. Moreover, while the questions in this paper dealt primarily with the framing of concepts in political debates, we believe that this method can be extended to facilitate answering a wide range of questions regarding the representation of meaning in texts.

Nevertheless, it is should be noted that this method, like all purely co-occurrence based ones, has some important limitations. While they can be used to identify broad thematic relationships between words, they are generally too blunt a tool for the analysis of more fine-grained semantic distinctions. For instance, both synonyms and antonyms of a word will produce vectors that are highly similar to that of the original word – in the senate corpus the terms *legal* and *illegal* are highly correlated (*r*=.82) because they both appear in very similar contexts relating to law and immigration. Consequently, when choosing terms for disambiguation and the identification of frames it is important to choose terms and frames that belong to different *semantic fields* and not simply polar opposites in the semantic meaning.

It is also important to remember that the method described in this paper does not replace methods that provide in-depth and detailed analysis, such as the manual examination of particular contexts. Instead it is intended to provide a means to conduct hypothesis testing based on corpus data. As such, it provides a large-scale overview of whether certain patterns in a dataset match those hypothesized by the research. Nevertheless, future developments of this method could employ measures such as cosine similarity to *identify* uses of a word that are markedly different and worthy of additional scrutiny.

With these caveats, we believe that the method presented in this paper has important advantages over alternative forms of analysis, and the analysis of simple co-occurrence patterns in particular. One such advantage is due to the focus of the method on the comparison of semantic fields rather than specific word types. This allows for analyses that are relatively stable regardless of the specific terms chosen. For example, the analysis of *terror* presented in this paper contrasted the two frames of *war* and *crime*. However, these terms can easily be replaced with related terms such as *military* and *trial*, respectively, for which the analysis would produce similar results. Stability across a range of terms suggests that the results obtained are indicative of more than just changes in simple patterns of word co-occurrence, but rather that the differences identified correspond to changes in overall patterns of discourse, word use, and meaning.

## Supporting Information

Text S1 Implementation details. (DOC)

Movie S1 2-dimensional visualization of the context vectors for *terror*, *war*, and *crime*. 2-dimensional visualization of the context vectors for *terror*, *war*, and *crime* from 1990. Each frame represents the context vectors over a 12-month period up to (and including) the month given in the caption. Because of memory constraints, context vectors for the 3 terms were aggregated by speaker for each month. This aggregated set of vectors was then used as a whole to compute a 2-dimensional MDS space, and plotted as a yearly running average on a month-by-month basis using the ggplot2 package for R [31].
(AVI)
