# Correction: Identifying Issue Frames in Text

**DOI:** 10.1371/annotation/f6708258-1101-40f2-9a91-be36b1b1afd8

**Published:** 2014-01-24

**Authors:** Eyal Sagi, Daniel Diermeier, Stefan Kaufmann

Several errors occurred in the DOI (Digital Object Identifier) links for the manuscripts listed in the Reference section. Please see an updated Reference list with the correct links below: 

References

1. Plous S (1993) The psychology of judgment and decision making. New York: McGraw-Hill. 302 p.

2. Tversky A, Kahneman D (1981) The Framing of decisions and the psychology of choice. Science 211: 453–458.

3. Schattschneider EE (1960) The Semi-Sovereign People. Hinsdale, IL: Dryden Press. 147p.

4. Lakoff G (2004) Don't Think of an Elephant: Know Your Values and Frame the Debate: The Essential Guide for Progressives. White River Jct., VT: Chelsea Green Publishing. 144 p.

5. Chong D, Druckman JN (2007) Framing Theory. Annual Review of Political Science 10: 103-126. doi: http://dx.doi.org/10.1146/annurev.polisci.10.072805.103054

6. Baumgartner FR, De Boef SL, Boydstun AE (2008) The Decline of the Death Penalty and the Discovery of Innocence. New York: Cambridge University Press

7. Druckman JN, Jacobs LR (2009) Presidential Responsiveness to Public Opinion. In: Edwards GC III, William G, editors. The Oxford Handbook of the American Presidency. Oxford: Oxford University Press. pp. 160–181.

8. Shapiro RY (2011) Public Opinion and American Democracy. Public Opin Q 75: 982–1017. doi: http://dx.doi.org/10.1093/poq/nfr053 

9. Baumgartner FR, Jones BD (1993) Agendas and Instability in American Politics. Chicago: University of Chicago Press. 298 p.

10. Baumgartner FR, Jones BD (2002) Policy Dynamics. Chicago: University of Chicago Press. 360 p.

11. Monroe BL, Schrodt PA (2008) Introduction to the Special Issue: The Analysis of Political Text. Polit Anal 16: 351-355. doi: http://dx.doi.org/10.1093/pan/mpn017 

12. Quinn KM, Monroe BL, Colaresi M, Crespin MH, Radev DR (2010) How to Analyze Political Attention with Minimal Assumptions and Costs. Am J Pol Sci 54: 209-228. doi: http://dx.doi.org/10.1111/j.1540-5907.2009.00427.x 

13. Yu B, Kaufmann S, Diermeier D (2008) Classifying party affiliation from political speech. Journal of Information Technology and Politics 5: 33-48. doi: http://dx.doi.org/10.1080/19331680802149608 

14. Yu B, Diermeier D, Kaufmann S, Godbout JF (2012) Language and Ideology in Congress. Br J Polit Sci 42: 31-55.

15. Firth, J (1957) Papers in Linguistics, 1934-1951. London: Oxford University Press. 233 p. http://www.worldcat.org/title/papers-in-linguistics-1934-1951/oclc/32594232 

16. Deerwester S, Dumais ST, Furnas GW, Landauer TK, Harshman R (1990) Indexing by Latent Semantic Analysis. Journal of the American Society for Information Science 41: 391-407. doi: http://dx.doi.org/10.1002/(SICI)1097-4571(199009)41:6%3C391::AID-ASI1%3E3.0.CO;2-9 

17. Landauer TK, Dumais ST (1997) A solution to Plato's problem: The Latent Semantic Analysis theory of the acquisition, induction, and representation of knowledge. Psychol Rev 104: 211-240. doi: http://dx.doi.org/10.1037/0033-295X.104.2.211 

18. Schütze H (1997) Ambiguity in language learning: computational and cognitive models. Chicago: University of Chicago Press. 176 p.

19. Levin E, Sharifi M, Ball J (2006) Evaluation of utility of LSA for word sense discrimination. In: Moore RC, Bilmes JA, Chu-Carroll JC, Sanderson M, editors. Proceedings of the 2006 Human Language Technology Conference of the NAACL, Companion Volume: Short Papers. Stroudsburg, PA: Association for Computational Linguistics. pp. 77-80. 

20. Schütze H (1998) Automatic word sense discrimination. Computational Linguistics 24: 97-124. http://dl.acm.org/citation.cfm?id=972724

21. Marcu D (2003) Automatic Abstracting. In: Darke MA, editor. Encyclopedia of Library and Information. New York: Marcel Dekker. pp. 245-256.

22. Riedel E, Dexter SL, Scharber C, Doering A (2006) Experimental Evidence on the Effectiveness of Automated Essay Scoring in Teacher Education Cases. Journal of Educational Computing Research 35: 267-287. doi: http://dx.doi.org/10.2190/U552-M54Q-5771-M677 

23. Sagi E, Kaufmann S, Clark B (2009) Semantic Density Analysis: Comparing Word Meaning across Time and Phonetic Space. In: Basili R, Pennacchiotti M, editors. Proceedings of the EACL 2009 Workshop on GEMS: Geometrical Models of Natural Language Semantics. Stroudsburg, PA: Association for Computational Linguistics. pp. 104-111.

24. Iyengar S, Kinder DR (1987) News That Matters: Television and American Opinion. Chicago: University of Chicago Press.

25. Takayama Y, Flournoy R, Kaufmann S (1998) Information Mapping: Concept-Based Information Retrieval Based on Word Associations. Stanford, CA: CSLI Publications. 8 p.

26. Sandhaus E (2008) The New York Times Annotated Corpus. Philadelphia, PA: Linguistic Data Consortium. Available: http://www.ldc.upenn.edu/Catalog/catalogEntry.jsp?catalogId=LDC2008T19. Accessed 20 January 2013.

27. Infomap [Computer Software] (2007) Stanford, CA. Available: http://infomap-nlp.sourceforge.net/. [^] Accessed 20 January 2013.

28. Hu X, Cai Z, Wiemer-Hastings P, Graesser A, McNamara D (2007) Strength, Limitations, and Extensions of LSA. In: Landauer TK, McNamara D, Dennis S, Kintsch W, editors. The Handbook of Latent Semantic Analysis. Mahwah, NJ: Lawrence Erlbaum Associates. pp. 401-426.

29. Lakoff G (2008) The political mind: Why you can’t understand 21st-century politics with an 18th-century brain. New York: Viking. 292 p.

30. Gentzkow M, Shapiro JM (2010) What Drives Media Slant? Evidence from U.S. Daily Newspapers. Econometrica 78: 35-71. doi: http://dx.doi.org/10.3982/ECTA7195 

31. Wickham H (2009) ggplot2: elegant graphics for data analysis. New York: Springer.

