# Supplementary material for: Identifying Issue Frames in Text
Source: PLoS One. 2013 Jul 16;8(7):e69185. doi: 10.1371/journal.pone.0069185 (PMC3712954; doi:10.1371/journal.pone.0069185)
Supplement: Text S1 — (DOC) [file pone.0069185.s001.doc]

## Supplementary Materials and Methods

Our method is based on the WordSpace paradigm introduced by Hinrich Schütze [1][2]. WordSpace was originally inspired by Latent Semantic Indexing [3] and is part of a family of methods commonly subsumed under the label Latent Semantic Analysis (LSA) [4].

## Word Vectors

All varieties of LSA are based on the assumption that measures of semantic similarity or distance between words can be obtained by observing their co-occurrence patterns in large text corpora. Unlike traditional measures of collocational strength, which are typically defined for individual pairs of words, LSA derives similarity patterns globally for a large class of words or the entire lexicon. This is achieved by associating each word in the vocabulary with a word vector in a high-dimensional space. The most widely used measure of similarity between two words then isthe cosine between the associated word vectors.

The word vectors used in our analysis were obtained using the open-source *Infomap* software package developed at Stanford University. From the word types in the corpus, filtered by a *stoplist* to remove high-frequency function words and other uninformative items (we used the default stoplist provided by the Infomap package), we choose a *vocabulary* $V$ comprising the 20,000 most frequent non-stop words, and a set *C* of 1,000 *content words* consisting simply of the 50^th^ through 1049^th^ most frequent words (these parameters were chosen because they are the defaults used by Infomap and have proven useful in previous research). This purely frequency-based selection may seem arbitrary, but it has the advantage of not requiring any human supervision or prior knowledge of the corpus. For each word in the vocabulary we then derive a representation of its co-occurrence profile with the content words. Specifically, we assemble a $\left|V\right|\times\left|C\right|$ *co-occurrence matrix* each of whose rows represents a vocabulary item and each of whose columns is labeled by a content word. This use of a *term-term* matrix is the main distinguishing feature of the Infomap approach. Most other implementations of LSA rely instead on a *term-document* matrix in which columns are labeled by documents rather than content words. While term-document co-occurrence counts are appropriate for document retrieval, they are less suitable in building models of lexical semantics. In contrast, term-term co-occurrence counts allow for a more localized notion of “co-occurrence.”

Each cell $\left\langle w,c\right\rangle$ records the number of occurrences of *c* in a fixed window of ±15 words (less if a document boundary intervenes) around an occurrence of $w$ in the corpus. These counts are weighed by a *tf.idf* measure on *c* [5]. Here *tf* and *idf* stand for *term frequency* and *inverse document frequency*, respectively. The basic idea is to counterbalance the raw frequency of occurrence of a term by the number of documents in which it occurs, based on the assumption that terms whose occurrences are widely dispersed over the documents are less useful in making semantic distinctions. The formula used by Infomap is

$tf.idf(c)=tf(c)\times\left(\log(D+1)-\log(df(c))\right)$,

where *tf(c)* and *df(c)* are the term frequency and document frequency of column label $c$, and $D$ is the number of documents in the corpus. The square root is taken to attenuate the influence of outliers and approximate a normal distribution, and the vectors are normalized. The matrix is then subjected to *Singular Value Decomposition* to derive three matrices *U, Σ, V^*^* (Infomap uses the SVD implementation found in SVDPACKC) [6]. Only the left singular vectors in *U*, truncated to 100 dimensions, are used in the subsequent computations. Thus ultimately each vocabulary item is represented as a 100-dimensional vector.

## Context Vectors

The word vectors typically used in LSA applications correspond to word types (i.e., vocabulary items). In our analysis of framing we are generally interested in different uses of the same word across time, speaker, or other variables. As discussed in the paper, we take a cue from work on word sense disambiguation and use the context of an occurrence of a given word as a stand-in for its meaning on that particular occurrence. To this end, once the word vectors are in place, we make a second pass over the corpus to derive context vectors for each occurrence of our target words of interest (e.g., *terror*, *woman*). For each occurrence$w_{i}$of a target word$w\in V$, all occurrences of vocabulary items within a fixed window of ±15 words of$w_{i}$(less if a document boundary intervenes) are collected, the corresponding vectors are added up, and the resulting vector is normalized. These context vectors can then be compared to each other and to word vectors, again using the cosine as a measure of similarity. These pairwise cosine values are the data points in our statistical analyses.

## References

1. Schütze H (1997) *Ambiguity in Language Learning: Computational and Cognitive Models*. University of Chicago Press, Chicago IL. 176 p.
2. Schütze H (1998) Automatic word sense discrimination. *Computational Linguistics* 24:97-124.
3. Deerwester S, Dumais ST, Furnas GW, Landauer TK, Harshman R (1990) Indexing by Latent Semantic Analysis. *Journal of the American Society for Information Science* 41:391-407.
4. Landauer TK, McNamara DS, Dennis S, Kintsch W (2007) *Handbook of Latent Semantic Analysis*. Mahwah, NJ: Lawrence Erlbaum Associates. 532 p.
5. Manning C, Schütze H (1999) *Foundations of Statistical Natural Language Processing*. Boston, MA: MIT Press. 680 p.
6. Berry MW (1992) Large scale singular value computations. *International Journal of Supercomputer Applications* 6:13-49.
